# Impact of Nitridation on Bias Temperature Instability and Hard Breakdown Characteristics of SiON MOSFETs

**DOI:** 10.3390/mi14081514

**Published:** 2023-07-28

**Authors:** Stanislav Tyaginov, Barry O’Sullivan, Adrian Chasin, Yaksh Rawal, Thomas Chiarella, Camila Toledo de Carvalho Cavalcante, Yosuke Kimura, Michiel Vandemaele, Romain Ritzenthaler, Jerome Mitard, Senthil Vadakupudhu Palayam, Jason Reifsnider, Ben Kaczer

**Affiliations:** IMEC, Kapeldreef 75, 3001 Leuven, Belgium; barry.osullivan@imec.be (B.O.); adrian.chasin@imec.be (A.C.); yaksh.rawal@imec.be (Y.R.); thomas.chiarella@imec.be (T.C.); camila.cavalcante@imec.be (C.T.d.C.C.); yosuke.kimura@imec.be (Y.K.); michiel.vandemaele@imec.be (M.V.); romain.ritzenthaler@imec.be (R.R.); jerome.mitard@imec.be (J.M.); senthil.vadakupudhupalayam@imec.be (S.V.P.); jason.reifsnider@imec.be (J.R.); ben.kaczer@imec.be (B.K.)

**Keywords:** bias temperature instability, hard breakdown, nitridation, nitrogen content, nitrided oxide, ramped voltages stress, SiON, SiO_2_, transistor lifetime, defects

## Abstract

We study how nitridation, applied to SiON gate layers, impacts the reliability of planar metal-oxide-semiconductor field effect transistors (MOSFETs) subjected to negative and positive bias temperature instability (N/PBTI) as well as hard breakdown (HBD) characteristics of these devices. Experimental data demonstrate that p-channel transistors with SiON layers characterized by a higher nitrogen concentration have poorer NBTI reliability compared to their counterparts with a lower nitrogen content, while PBTI in n-channel devices is negligibly weak in all samples independently of the nitrogen concentration. The Weibull distribution of HBD fields extracted from experimental data in devices with a higher N density are shifted towards lower values with respect to that measured in MOSFETs, and SiON films have a lower nitrogen concentration. Based on these findings, we conclude that a higher nitrogen concentration results in the aggravation of BTI robustness and HBD characteristics.

## 1. Introduction

The breath-taking development of micro- and nanoelectronics relies on the rapid scaling of the metal–oxide–semiconductor field effect transistor (MOSFET) based on silicon. To meet requirements dictated by mobile/portable electronic products and applications for long battery lifetimes and hence reduced OFF-currents and optimized ON/OFF-current ratio, novel transistor architectures—starting from planar devices and going to 3D geometries such as finFETs [1,2] and continuing to nanosheet [3,4], forksheet [5,6,7], and complementary FETs [8,9]—have been introduced. However, before the launching of any new transistor node, its reliability characteristics should be carefully assessed together with the performance, power consumption, etc. Among reliability issues featured by modern MOSFETs, the most detrimental ones are bias temperature instability (BTI) [10,11], time-dependent dielectric breakdown/hard breakdown (TDDB/HBD) [12,13], and hot-carrier degradation (HCD) [14,15,16]. Although HCD has been repeatedly flagged as a very severe degradation concern in confined 3D transistor architectures [17,18], it lies outside the scope of this paper. The reason for that is that BTI and HBD are intimately linked with the gate stack quality, while HCD is rather related to carrier acceleration by the electric field, which is determined by the voltage partition in the transport (i.e., source-drain) direction [19,20,21,22,23,24]. Therefore, rather than focusing on the device architecture optimization aiming to mitigate HCD, the focus of this work is put on optimization of SiON layers to suppress such reliability issues as BTI and HBD.

Among various techniques aimed at alleviating BTI and improving breakdown characteristics of the gate stack, nitridation of the gate dielectric layers has been the subject of extensive studies performed by several groups. Regarding the impact of nitridation on TDDB/HBD, to the best of our knowledge, there are a very limited number of publications addressing this subject. Among them, are the papers by Joo et al. [25] and Mazumder et al. [26], which analyzed how nitridation in NO${}_{2}$ ambient impacts TDDB and concluded that low-pressure nitridation results in the improvement of TDDB robustness, while nitridation at an increased pressure leads to poorer TDDB characteristics. A TDDB lifetime improvement due to annealing in the NO ambience was reported by Chen et al. [27], while Lee et al. [28] demonstrated that ex situ N annealing has a very weak impact on TDDB. Therefore, one can summarize these studies, and depending on nitridation conditions, this fabrication step can either improve TDDB/HBD robustness or aggravate it.

Very similar to the case of TDDB/HBD, a consensus regarding the impact of nitridation on BTI reliability has not been reached so far. On the one hand, several groups suggest that the incorporation of N into a gate stack suppresses BTI. Thus, O’Sullivan et al. demonstrated that N (or F) diffusion into the gate dielectric layer can shift the band of donor-like traps, thereby impeding negative BTI (NBTI) [29]. In [30], O’Connor et al. reported that N passivates traps in HfSiO layers but this behavior can be reversed during stress, thereby giving rise to NBTI and a stress-induced leakage current (which accompanies TDDB). Further, Maheta et al. [31] speculated that the impact of N on NBTI is very intricate and is comprised of changing properties of interface traps and oxide states, thereby resulting in different characteristic energies for trap activation and NBTI time exponents. Joshi et al. [32] published a reduction of BTI due to N incorporation. On the other hand, Garros et al. claimed that in their MOSFETs with high-*k*/metal gate stacks N results in severe aggravation of BTI reliability [33,34]. Their results are consistent with data published by Reisinger et al., which demonstrate stronger NBTI in nitrided oxides compared to non-nitrided ones [35]. Finally, Takasaki et al. [36] used the charge-pumping technique to resolve the interface trap density in devices with different parameters of the nitridation process subjected to BTI stress. Their results suggest that BTI aggravates transistors with a higher N content.

Renewed interest in BTI and HBD reliability of MOSFETs with nitrided gate oxides is related to the introduction of the forksheet transistor architecture, where n- and pFET sheets are separated by an Si${}_{3}$N${}_{4}$ (or SiON) wall. Recent experimental studies of reliability issues in forksheet FETs did not show extra charge trapping in the Si${}_{3}$N${}_{4}$ (or SiON) wall [37]. From the simulation perspective, this behavior was explained to be exclusively due to electrostatic reasons, i.e., the corresponding voltage partition in the forksheet wall [38]. However, for further boosting device reliability, it is important to understand whether N incorporation into ${\mathrm{SiO}}_{2}$ and SiON films improves or aggravates transistor robustness. Moreover, the knowledge on the reliability of SiON gate stacks acquired in this work should also be of relevance for novel FET architectures employing SiON related materials outside their gate stack, e.g., in the forksheet FET wall, transistor spacers, etc.

Therefore, the scope of this paper is an experimental investigation on the impact of nitridation of gate SiON layers on BTI and HBD characteristics. We also aim at contributing towards reconciling controversies regarding the impact of nitridation on gate oxide reliability.

## 2. Devices

We employed planar n- and p-channel MOSFETs fabricated in a 300 mm line using a standard gate first flow. These transistors have a gate stack made of an SiON layer and a 100 nm polySi layer deposited in situ. To fabricate the SiON layer, first an ${\mathrm{SiO}}_{2}$ film was grown by steam generation in a rapid thermal processing system and then this film was subjected to nitridation in the N${}_{2}$ ambient, followed by a post-nitridation anneal. We used two types of nitridation processes:

- The process of reference (POR), which includes plasma nitridation at a higher value of the radio frequency (RF) power of 1000 W under a pressure of 20 Torr for 12 s.
- The alternative process with softer nitridation (SN) conducted at a lower RF power of 900 W under a pressure of 20 Torr for 12 s.

Let us emphasize that these two transistor splits are close to each other, but we have a narrow device target window specified by the targeted equivalent oxide thickness (EOT), the gate leakage current density, etc. Therefore, in order to ensure that these splits fit into the specified window, we were limited in variations of the fabrication process parameters.

Devices with a targeted physical thickness of the SiON layer of 1.9 nm were fabricated using both the POR (this sample is labeled as “S #1”) and the process including the SN step (this sample is labeled as “S #2”), see Table 1. In addition to this, MOSFETs with a 2 Å thicker SiON layer (targeted physical thickness is 2.1 nm) were fabricated by the POR, (S #3, Table 1). Finally, with the SN-based process, transistors with a 2 Å thinner SiON film (targeted thickness is 1.7 nm, S #4 and 5) were also grown. The EOT values for all samples, obtained using capacitance-voltage measurements combined with the CVC model for EOT extraction [39], are summarized in Table 1. Within the EOT extraction procedure, the potential partition in the depleted poly-Si layer and correction due to quantum confinement at the Si/SiON interface were taken into account.

Threshold voltage values were extracted from transfer characteristics ($I_{d}-V_{g}$, where $I_{d}$ is the drain current and $V_{g}$ is the gate voltage) using the maximum transconductance method; see Table 1. From Table 1, it can be observed that although samples S #4 and S #5 have the same targeted thickness, which is 2 Å less than that of the POR device (sample S #1), in practice, their EOT is comparable to the EOT of the reference sample (sample S #1). In addition, their $V_{t}$ values substantially deviate from those of other samples. As a consequence, one can expect that S #4 and S #5 have an inferior reliability among the entire set of devices.

The extracted threshold voltage values are consistent with the parameters of the nitridation step. Indeed, N incorporated in the gate dielectric is known to result in positive charges distributed throughout the layer, which, in turn, induce a threshold voltage shift towards lower values. Samples #1 and 2 have the same targeted physical thickness, but, in the latter case, nitridation was carried out with lower power; therefore, S #2 has a lower N content compared to S #1. The corresponding $V_{t}$ voltages are consistent with this trend, i.e., $V_{t}$ values of S #2 are higher for both n- and pMOSFETs as compared to those of S #1. The wafers S #4 and 5 were fabricated by the same process as S #2, but the physical thickness of their SiON layers is the lowest among all devices used in this study; therefore, they have the highest concentration of nitrogen and hence the lowest values of $V_{t}$. Finally, for S #1 and 3, the same nitridation process was employed; however, the wafer S #3 has a 2 Å thicker SiON layer and therefore a lower N content and higher threshold voltages. A summary of N content can be found in the last column of Table 1, where a higher rank corresponds to a higher concentration, i.e., ‘1’ corresponds to the lowest N content and ‘4’ to the highest N concentration.

We would like to note that although a comparison of BTI and HBD characteristics of MOSFETs with SiON films against those acquired in devices with pure ${\mathrm{SiO}}_{2}$ as a gate dielectric would be informative, such a comparison was not performed in this study. The reason for that is that the reliability measurements were carried out to qualify the gate oxide integrity for the 65 nm technology node with polySi/SiON gate stack. Therefore, in this EOT range (see Table 1), it is required to employ SiON as high-*k* dielectric to ensure lower gate leakage and better electrostatic control of the channel. Consequently, we do not have ${\mathrm{SiO}}_{2}$-based MOSFETs at our disposal.

## 3. Experiment and Data Processing

In ultra-scaled FETs, characteristics of pristine devices can vary from sample-to-sample. This is called “time-zero variability” [40], which originates from several sources such as random dopant fluctuations, metal gate granularity, line edge roughness, fluctuations in material properties, oxide thickness variations, etc. [41,42,43,44,45,46]. Degradation of device characteristics is due to generation/activation of defects. Ultra-scaled FETs contain just a handful of defects (and their precursors), which are randomly distributed throughout a transistor; therefore, contributions of individual defects to degradation can be discernible. Thus, BTI and HCD types of stress leads to time-dependent variability in small-area MOSFETs [47,48,49]. In contrast, in large-area devices, BTI/HCD is due to a collective response of a very large number of defects, and a sample-to-sample scattering of degradation characteristics is not prominent. Hence, in order to avoid BTI-induced variability, we intentionally chose large-area devices. Regarding TDDB/HBD, this type of degradation is driven by defect build-up across the dielectric film resulting in the formation of a percolation path. Hence, for the processing/interpretation of experimental HBD data, stochastic approaches are typically employed for both large- and small-area devices [12,50]. To summarize, for BTI and HBD measurements, we chose MOSFETs with a gate length ($L_{g}$) and a width (*W*) of 1 μm.

The studied samples were subjected to ramped voltage stress (RVS) in BTI and HBD regimes at room temperature. For both degradation modes, we used the RVS technique developed by Kerber et al. [51,52]. Note that for BTI, the extended measure–stress–measure procedure (eMSM) [53] is commonly employed, while for HBD studies, the standard routine is constant voltage stress (CVS) [54,55,56]. Despite the fact that the eMSM and CVS techniques provide a more comprehensive insight into BTI and HBD, respectively, the RVS scheme was proven to have same accuracy for both BTI [51,52] and TDDB/HBD [57] degradation modes. On the other hand, the RVS technique allows us to dramatically reduce the experimental time, thereby making itself the routine of choice for conducted BTI and HBD investigations.

In these experiments, n-channel transistors were stressed under positive gate bias $V_{g}$ (in both BTI and HBD regimes), while their p-channel counterparts were subjected to RVS stress at negative $V_{g}$.

### 3.1. Bias Temperature Instability

**Figure 1:**
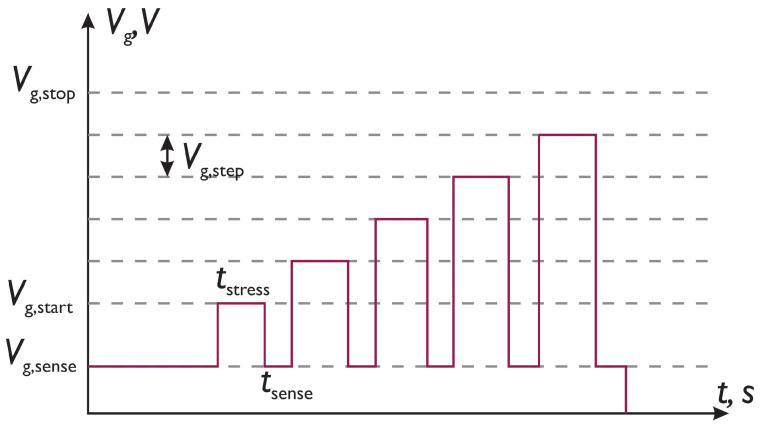
Schematic representation of RVS BTI measurements.

The RVS measurement procedure for BTI, which is comprised of sense and stress phases, is schematically depicted in Figure 1. Prior to BTI stress, $I_{d}-V_{g}$ curves in a limited $V_{g}$ range (we used $|V_{g}|$ sweeping the range of [0; 1.0] V) of pristine transistors were measured. $I_{d}-V_{g}$ characteristics were then employed to extract the $V_{t}$ value and the corresponding drain current $I_{t}$ = $I_{d}\;\left(V_{g}=V_{t}\right)$. Next, we applied a stress pulse of a duration of $t_{\mathrm{stress}}$ with an increasing amplitude: for the stress phase *i*, the signal amplitude is $V_{\text{g,start}}+i\times V_{\text{g,step}}$. These ramps sweep the voltage range of [$V_{\text{g,start}};V_{\text{g,stop}}$], increasing the amplitude each time by $V_{\text{g,step}}$, see Figure 1. Each stress phase labeled with *i* is followed by a phase (with the same index *i*) when we interrupt the stress and extract $V_{t}$.

**Figure 2:**
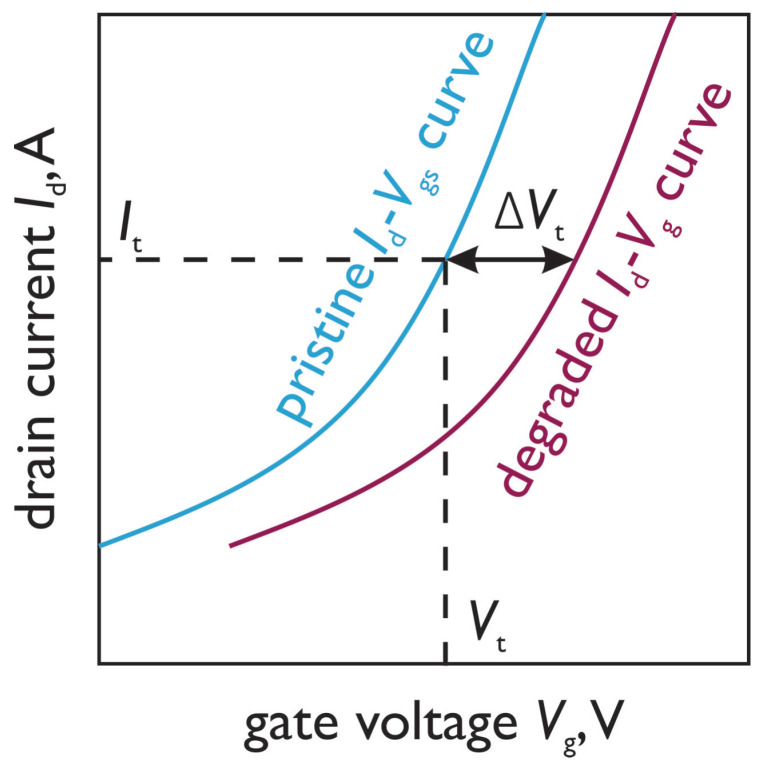
Schematic representation of the $\Delta V_{t}$ extraction technique. First, the $I_{d}-V_{g}$ curve of the pristine device is measured, thereby allowing the extraction of $V_{t}$ and $I_{t}$. Next, at each stress time step *t*, $V_{t}\left(t\right)$ is determined as the gate voltage at which $I_{d}\left(V_{g}\right)=I_{t}$ and therefore $\Delta V_{t}\left(t\right)$ = $V_{t}\left(t\right)-V_{t}\left(0\right)$.

$V_{t}$ is determined as the gate voltage which satisfies the criterion: $I_{t}$ = $I_{d}\left(V_{g}\right)$; see Figure 2. The obtained value is $V_{t}\left(t\right)$, with *t* being the cumulative stress time: $t\approx i\times t_{\mathrm{stress}}$. Therefore, the stress-induced shift in the threshold voltage is determined as $\Delta V_{t}$ = |$V_{t}\left(t\right)-V_{t}\left(0\right)$|. Another important parameter of the RVS routine is the ramp rate $RR=V_{\text{g,step}}/t_{\mathrm{stress}}$. It is noteworthy that a lower RR, ceteris paribus, results in a larger $\Delta V_{t}$. This is because the stress time at each voltage step is longer for the slower ramp rate (i.e., if the $V_{\text{g,step}}$ value is constant and $t_{\mathrm{stress}}$ is longer, then their ratio $V_{\text{g,step}}/t_{\mathrm{tstress}}$, defined as RR, is lower). For each RR value and type of MOSFETs (i.e., n- and p-channels and the fabrication process flow) we used eight samples.

For NBTI measurements, we used the following parameters: $V_{\text{g,start}}=V_{t}-0.4$ V, $V_{\text{g,stop}}=V_{t}-4.0$ V; the voltage step $V_{\text{g,step}}$ and the pulse duration $t_{\mathrm{stress}}$ were chosen to ensure five different ramp rates of 0.1, 0.3, 1.0, 3.0, and 7.5 V/s. The drain voltage $V_{d}$ was set to −0.05 V. PBTI measurements were conducted in a similar fashion with $V_{\text{g,start}}=V_{t}+0.4$ V, $V_{\text{g,stop}}=V_{t}+4.0$ V, and RR = 0.1, 0.3, 1.0, 10.0, and 3.3 V/s; $V_{d}$ = 0.05 V.

To process BTI data acquired using the RVS routine, we assume the following $\Delta V_{t}$ dependency on the stress time and gate bias:

(1) $$\Delta V_{t}\approx A_{0}{\left(\frac{|V_{g}-V_{t}\left(0\right)|}{t_{\mathrm{ox}}}\right)}^{\gamma }t^{n}=A_{0}{\left(\frac{|V_{\mathrm{ov}}|}{t_{\mathrm{ox}}}\right)}^{\gamma }t^{n}=A_{0}E_{\mathrm{ov}}^{\gamma }t^{n},$$

where $A_{0}$ is a prefactor (accounting for the thermal activation of the BTI process), $t_{\mathrm{ox}}$ is the EOT of the gate dielectric layer, $V_{\mathrm{ov}}=V_{g}-V_{t}$ is the overdrive voltage, $E_{\mathrm{ov}}=V_{\mathrm{ov}}/t_{\mathrm{ox}}$, while *n* and γ are exponents determining time dependency and field acceleration of BTI, respectively. According to Kerber et al. [51,52], this formula can be rewritten in a manner to link $\Delta V_{t}$ to the ramp rate RR:

(2) $$\Delta V_{t}=\frac{A_{0}}{{\left(\frac{\gamma }{n}+1\right)}^{n}}\;E_{\mathrm{ov}}\;RR^{-n}.$$

Therefore, if the threshold voltage shift was measured as a function of $V_{\mathrm{ov}}$/$E_{\mathrm{ov}}$ and the RR, its time dependency can be extracted.

### 3.2. Hard Breakdown

In case of HBD, we used the scheme very similar to the RVS BTI measurement routine. However, instead of the sense phase, we monitored the gate leakage current $I_{g}$. In order to detect an HBD event, which manifests itself by an abrupt increase in the gate current observed at a stress voltage $V_{\mathrm{BD}}$, we aimed at sweeping the $\left[V_{\text{g,start}};V_{\text{g,stop}}\right]$ range with the minimum $V_{\text{g,step}}$ available at our measurement setup. Therefore, for HBD in nMOSFETs, we employed $V_{\text{g,start}}=0.0$ V, $V_{\text{g,stop}}=5.0$ V, and $V_{\text{g,step}}=0.01$ V; for the ramp rate, we used a single value of RR = 0.09 V/s. pMOSFETs were stressed at $V_{\text{g,start}}=0.0$ V, $V_{\text{g,stop}}=-5.0$ V. In all cases $V_{d}$ = 0 V. For acquiring a comprehensive statistical set required for the extraction of the breakdown voltage distribution, we used 48 samples for each channel polarity and type of transistors (summarized in Table 1).

Extracted $V_{\mathrm{BD}}$ values are then binned into a Weibull distribution. First, using the Bénard approximation [58], we calculate probabilities $F_{i}=F_{i}\left(V_{\mathrm{BD},i}\right)$ and then convert them to weibits $W_{i}$. Next, we fit the $W(V_{\mathrm{BD}})$ dependency with a Weibull distribution and extract its parameters β (the shape factor) and η (the scale factor). To achieve this goal, we use the maximum likelihood estimation method.

## 4. Results and Discussion

### 4.1. Bias Temperature Instability

**Figure 3:**
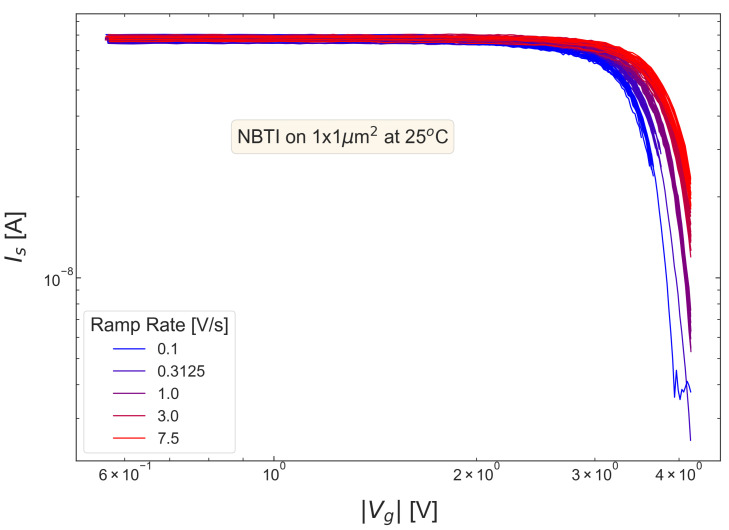
The typical evolution of the source-drain current $I_{s}$ during RVS NBTI stress. These current values were measured during sense phases. One can observe that $I_{s}$ decreases with the stress voltage; this decrease is a manifestation of device degradation during NBTI. At a lower ramp rate, the $I_{s}$ change is more pronounced. The data are shown for the SN pMOSFET with the targeted physical thickness of 1.9 nm (S #2).

In RVS BTI measurements, at each sense phase, we recorded the drain current $I_{d}$ and plotted $I_{d}$ values as a function of stress voltage $V_{g}$. An example of these $I_{d}$ traces obtained during NBTI stress for the pMOSFET fabricated using soft nitridation and with the targeted SiON thickness of 1.9 nm (wafer S #2) is given in Figure 3. The degradation of the device performance manifests itself by $I_{d}$ reduction, and, this reduction, as discussed before, becomes more pronounced at lower ramp rates. The recorded $I_{d}$ dependencies allowed us to extract threshold voltage shifts $\Delta V_{t}$ for each sense phase.

**Figure 4:**
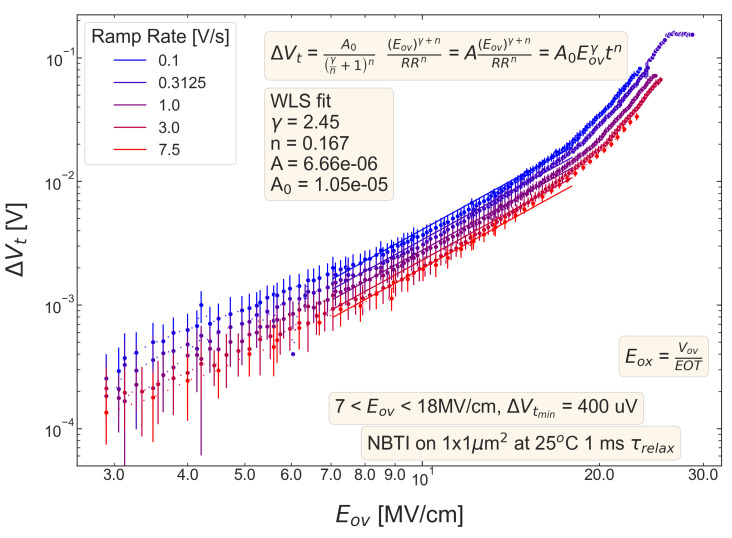
S #1 (POR), pMOSFETs: the threshold voltage shift $\Delta V_{t}$ caused by NBTI in pMOSFETs as a function of the overdrive oxide field $E_{\mathrm{ov}}$. The experimental $\Delta V_{t}\left(E_{\mathrm{ov}}\right)$ dependency is reproduced by Equation (1).

**Figure 5:**
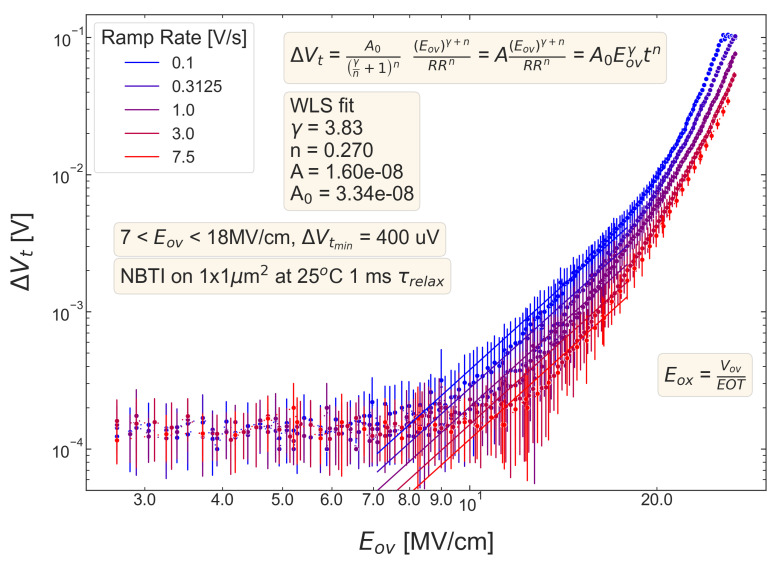
The same as Figure 4 but for S #2 (SN).

**Figure 6:**
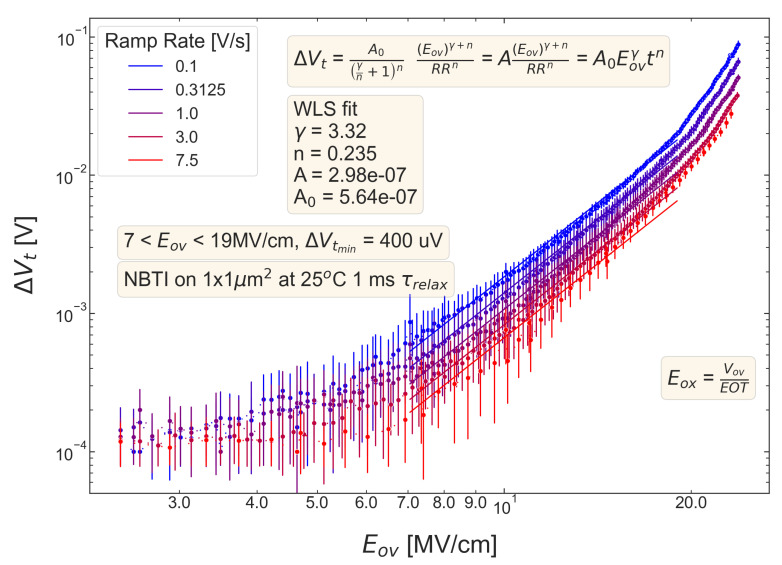
The same as Figure 4 but for S #3 (POR + 2 Å).

**Figure 7:**
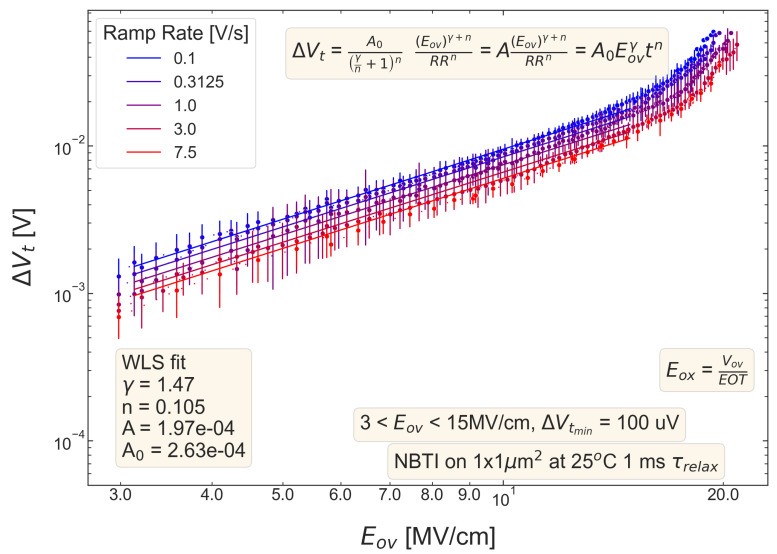
The same as Figure 4 but for S #4 (SN − 2 Å).

**Figure 8:**
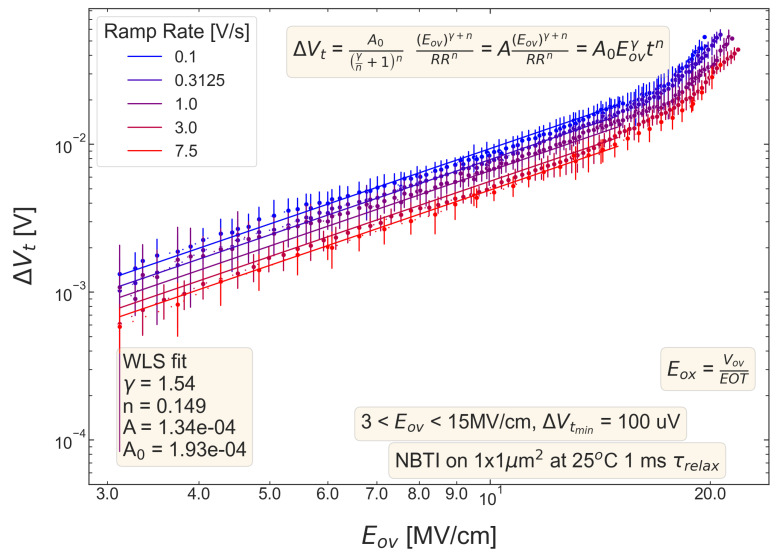
The same as Figure 4 but for S #5 (SN − 2 Å).

For all five pMOSFETs subjected to *NBTI*, dependencies of the threshold voltage $\Delta V_{t}$ shift on $E_{\mathrm{ov}}=V_{\mathrm{ov}}/t_{\mathrm{ox}}$ are shown in Figure 4, Figure 5, Figure 6, Figure 7 and Figure 8. $\Delta V_{t}$ changes are mean values obtained by averaging over the entire ensemble of eight $\Delta V_{t}\left(E_{\mathrm{ov}}\right)$ curves measured at a given RR rate. In Figure 4, Figure 5, Figure 6, Figure 7 and Figure 8, the error bars for threshold voltage changes are also shown; we conclude that although standard deviations of $\Delta V_{t}\left(E_{\mathrm{ov}}\right)$ values are discernible, they are small. One can observe that $\Delta V_{t}$ values become larger at lower ramp rates and this trend agrees with Equation (2). Another pronounced peculiarity is that at high electric fields of $E_{\mathrm{ov}}∼20$ MV/cm, the slope of $\Delta V_{t}\left(E_{\mathrm{ov}}\right)$ dependencies increases; this behavior is typical for all considered samples. Such a peculiarity is most probably related to non-equilibrium BTI driven by electrons traveling in the SiON conduction band, which can gain substantially high energies due to acceleration by the electric field and induce hydrogen release at the SiON/Si interface. Generation of interface traps according to this scenario was reported by the group from IBM [59,60] and recently by Bastos et al. [61]. However, this process occurs at very high electric fields and is related to another physical mechanism than that responsible for “conventional” BTI. Hence, we do not consider this degradation scenario while extracting device time-to-failure (TTF). It is worth mentioning that BTI measurements continued up to high $V_{g}$ values, and before reaching these voltages most of the devices broke down. During the processing of BTI data, we applied a filtering of HBD-induced current jumps; therefore, HBD events are not pronounced in Figure 4, Figure 5, Figure 6, Figure 7 and Figure 8.

Experimental $\Delta V_{t}\left(E_{\mathrm{ov}}\right)$ curves were captured by Formula (2) and the exponents γ and *n* were extracted; from Figure 4, Figure 5, Figure 6, Figure 7 and Figure 8, it can be observed that the agreement is good. For the fitting procedure, we employed the weighted linear regression method. Note that under a considerable threshold voltage shift, we assume $\Delta V_{t}≳1$ mV; therefore, $V_{t}$ values below this level were down-weighted. This also resulted in a more narrow—compared to the range determined by the used $V_{\text{g,start}}$ and $V_{\text{g,stop}}$ values (Figure 1)–$E_{\mathrm{ov}}$ range. For instance, one can observe that for S #2 (Figure 5) we carried out fitting for $E_{\mathrm{ov}}\geq 7$ MV/cm because at lower fields the threshold voltage shift is below 1 mV, i.e., negligibly small. In contrast, NBTI demonstrated by samples S #4 and 5 is much more significant; therefore, we could fit experimental data without limiting the $E_{\mathrm{ov}}$ range at the low value side. We also avoided the aforementioned $E_{\mathrm{ov}}$ region with a higher slope of the $\Delta V_{t}\left(E_{\mathrm{ov}}\right)$ curve (e.g., for S #1, this change is visible at $E_{\mathrm{ov}}∼17$ MV/cm, see Figure 4). Considering this $E_{\mathrm{ov}}$ range would result in an overestimated TTF value, which is determined as a stress time at which $\Delta V_{t}$ reaches a margin of 30 mV. This is because we extract the device lifetime by extrapolating the dependency of $\Delta V_{t}$ on $V_{\mathrm{ov}}$ (or $E_{\mathrm{ov}}$) from the experimentally available range to cover the operating voltage ($V_{\mathrm{dd}}$) value. However, a steeper $\Delta V_{t}\left(V_{\mathrm{ov}}\right)$ curve would result in a smaller $\Delta V_{t}\left(V_{\mathrm{dd}}\right)$ shift and hence larger TTF.

From Figure 4, Figure 5, Figure 6, Figure 7 and Figure 8, one can observe, that, as expected (see Section 2), devices S #4 and 5 have poor NBTI characteristics, i.e., relatively high $\Delta V_{t}$ values already at a low $E_{\mathrm{ov}}$; the most gradual slope of $\Delta V_{t}\left(E_{\mathrm{ov}}\right)$ curves determined by the parameter γ. On the contrary, the MOSFET S #2, which received soft nitridation and has a larger physical thickness of SiON than S #4 and 5, features the best NBTI reliability throughout the entire sample selection: negligibly small $\Delta V_{t}$ shift up to $E_{\mathrm{ov}}∼8$ MV/cm and the largest value of the exponent γ. If we compare S #1 and S #3, which have the same process flow but differ in the targeted physical thickness, one can observe that the latter one (with a lower N content) has substantially better NBTI reliability. Based on data presented in Figure 4, Figure 5, Figure 6, Figure 7 and Figure 8, we conclude that MOSFETs with a lower N concentration (see Table 1) in their SiON layers demonstrate superior NBTI behavior.

**Figure 9:**
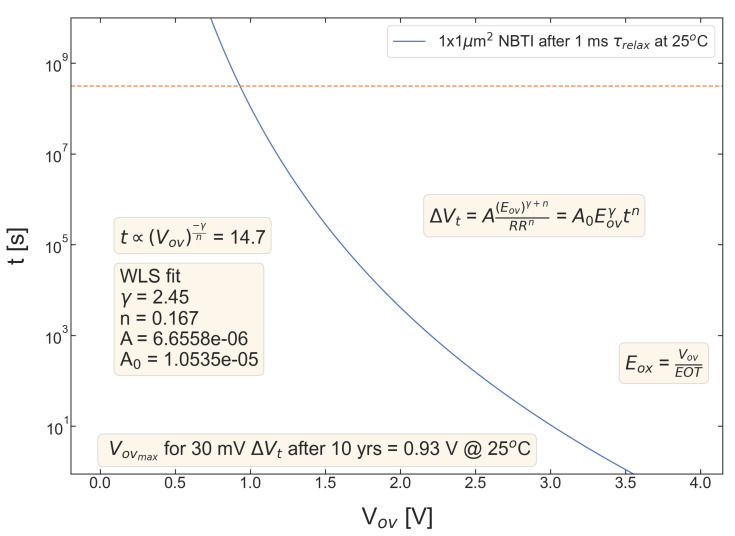
S #1 (POR), pMOSFETs: device time-to-failure (corresponding to the threshold voltage shift of $\Delta V_{t}=30$ mV) as a function of the overdrive voltage $V_{\mathrm{ov}}$. The upper limit of the safe operating area is determined as the $V_{\mathrm{ov}}$ corresponding to TTF of 10 years.

**Figure 10:**
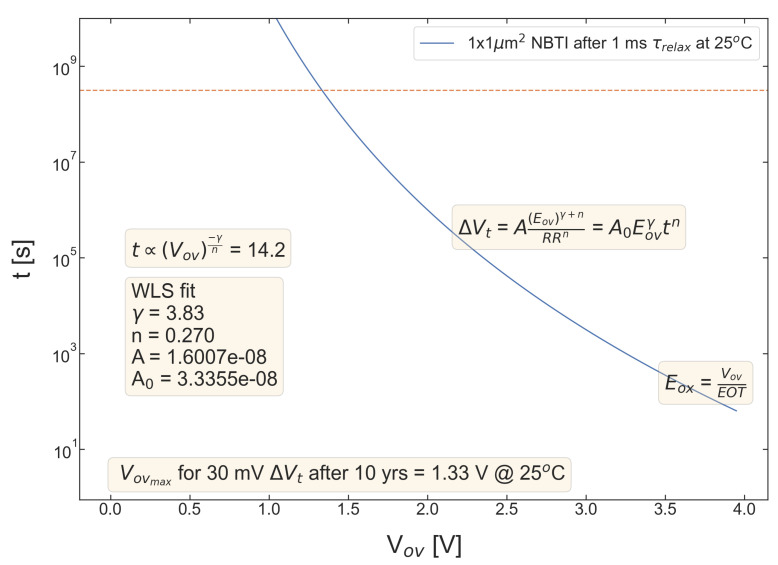
The same as Figure 4 but for S #2 (SN).

**Figure 11:**
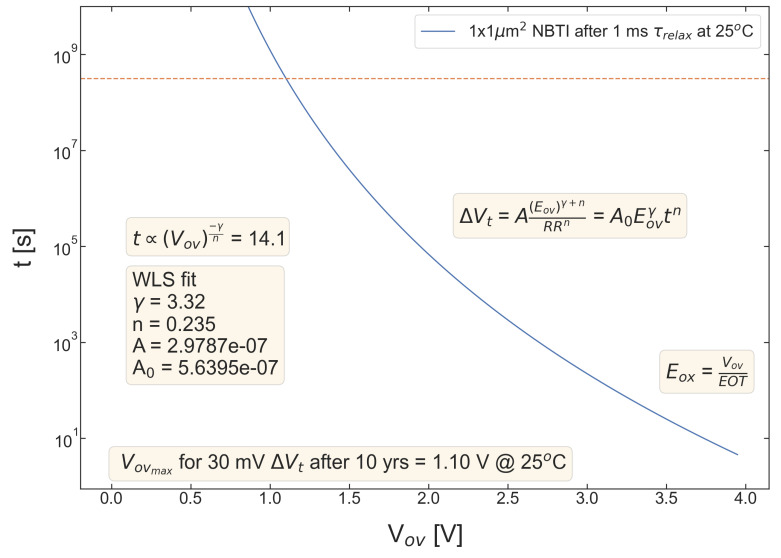
The same as Figure 4 but for S #3 (POR + 2 Å).

**Figure 12:**
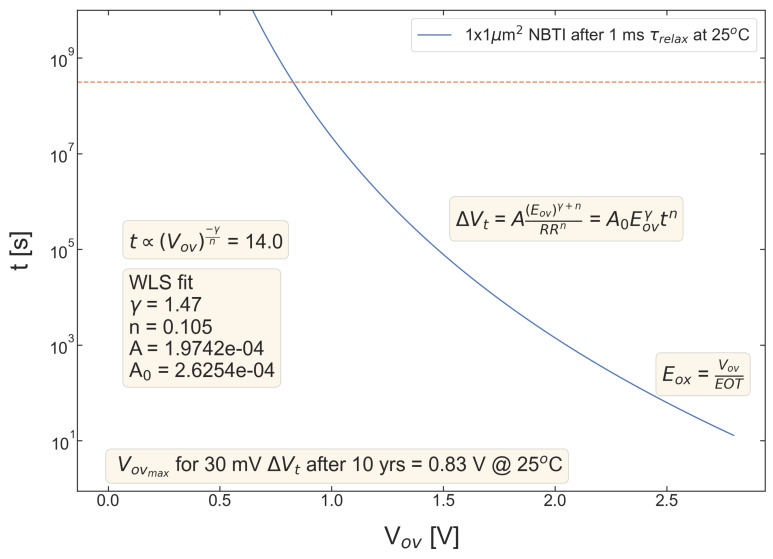
The same as Figure 4 but for S #4 (SN − 2 Å).

**Figure 13:**
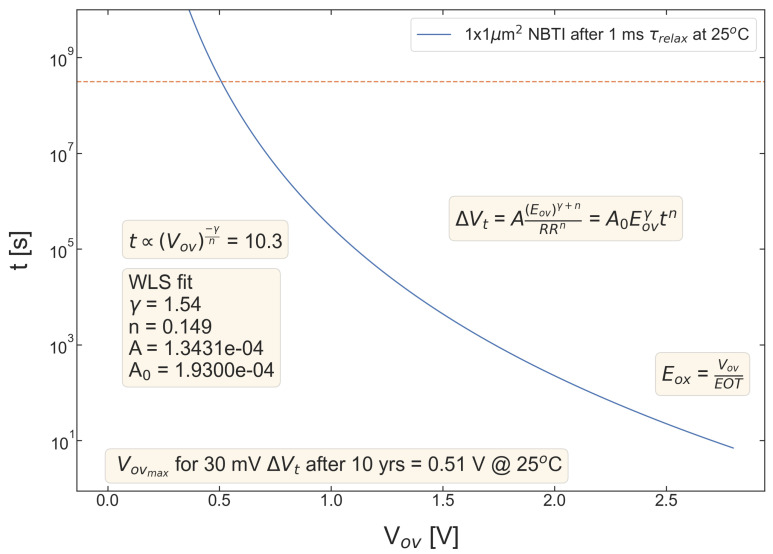
The same as Figure 5 but for S #5 (SN − 2 Å).

Figure 9, Figure 10, Figure 11, Figure 12 and Figure 13 summarize stress time values *t* corresponding to $\Delta V_{\mathrm{th}}=30$ mV plotted as a function of $V_{\mathrm{ov}}$ for all devices. These curves allow us to determine the upper limit of the safe operating area of these devices, as the $V_{\mathrm{ov}}$ value of each device lifetime is equal to 10 years. As consistent with the data set from Figure 4, Figure 5, Figure 6, Figure 7 and Figure 8, S #4 and 5 have the lowest $V_{\mathrm{ov}}\left(t=10\;\mathrm{yrs}\right)$ voltages, while S #2 is again the winner. More general, MOSFETs with a lower nitrogen content have longer TTF values.

**Figure 14:**
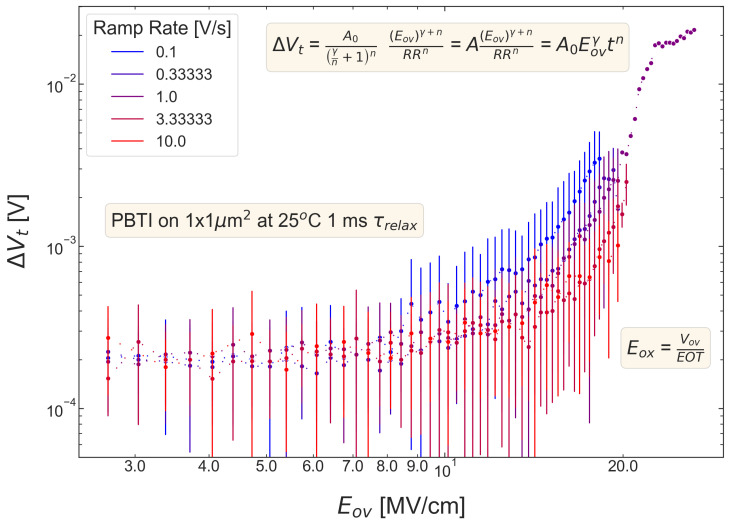
$\Delta V_{t}\left(E_{\mathrm{ov}}\right)$ dependencies measured during PBTI stress applied to the MOSFET which was fabricated by the POR process and has the targeted SiON thickness of 1.9 nm (S #1). One can observe that at the highest $E_{\mathrm{ov}}$ value of ∼20 MV/cm, the threshold voltage shift hardly reaches 4 mV; therefore, we can conclude that PBTI is negligibly weak. The same holds true for all other devices studied in this work (data sets for other devices are not shown in the paper).

*PBTI* stress was applied to nMOSFETs. Figure 14 summarizes $\Delta V_{t}\left(E_{\mathrm{ov}}\right)$ curves obtained using samples from the wafer with the SiON film, which was grown by the POR and has the targeted thickness of 1.9 nm (S #1). It is pronounced that at low electric fields, $\Delta V_{t}$ values hardly overcome the noise level, and values measured at different ramp rates are not discernible. Even at such a high field as $E_{\mathrm{ov}}=20$ MV/cm, the threshold voltage shift reaches a level of 4 mV, thereby suggesting that PBTI is very weak. Due to this reason, we perform neither a fitting of $\Delta V_{t}\left(E_{\mathrm{ov}}\right)$ with Equation (2) nor an extraction of the $V_{\mathrm{ov}}$ corresponding to TTF of 10 years at the margin of $\Delta V_{t}=30$ mV. Let us emphasize that this behavior is typical for all samples examined in this work (data are not shown). Therefore, we can conclude that in all these nMOSFETs, the PBTI is negligibly weak; therefore, we cannot judge which samples demonstrate superior PBTI reliability.

Note that weak PBTI in nMOSFETs is very typical for transistors with silicon oxides/nitrides [10]. This can be explained assuming that the physical mechanism underlying BTI is the capture/emission of charge carriers by/from traps in the dielectric layer [11], and, in the case of PBTI in n-channel FETs with ${\mathrm{SiO}}_{2}/\mathrm{SiON}/\mathrm{SiN}$ gate dielectric layers, the energetical position of the trap band is so that these traps are not accessible for the carriers (which are inversion electrons in the case of PBTI in nMOSFETs) [62].

### 4.2. Hard Breakdown

**Figure 15:**
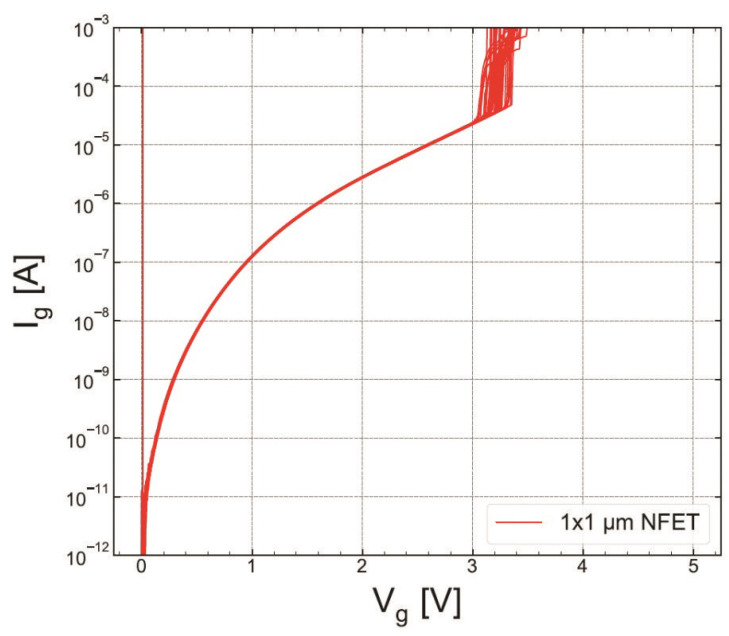
Gate current $I_{g}$ as a function of stress voltage $V_{g}$ recorded during RVS HBD measurements conducted on the POR nMOSFET with the targeted SiON thickness of 1.9 nm (sample S #1).

**Figure 16:**
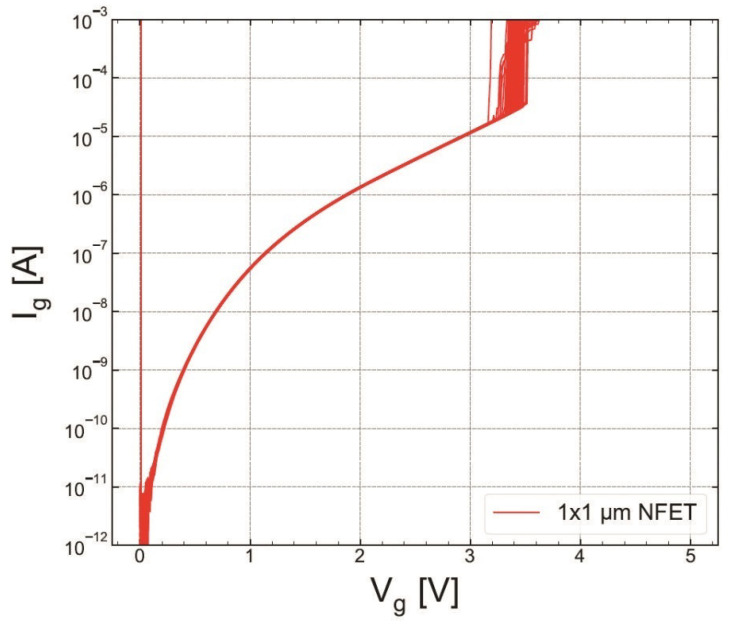
The same as Figure 15, but for the nMOSFET which received softer nitridation with the targeted SiON thickness of 1.9 nm (sample S #2).

**Figure 17:**
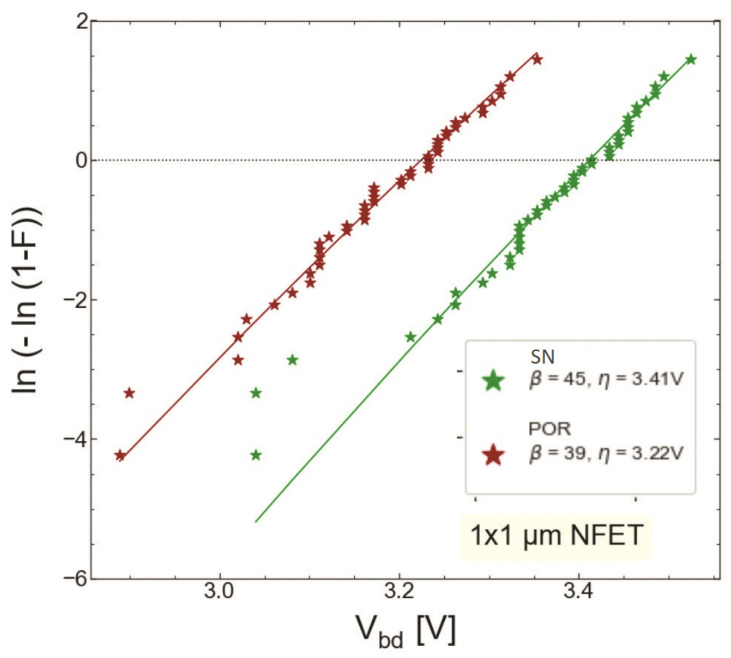
Weibit plots of breakdown voltages for the nMOSFETs fabricated by the POR (S #1, red symbols) and those exposed to SN (S #2, green symbols).

**Figure 18:**
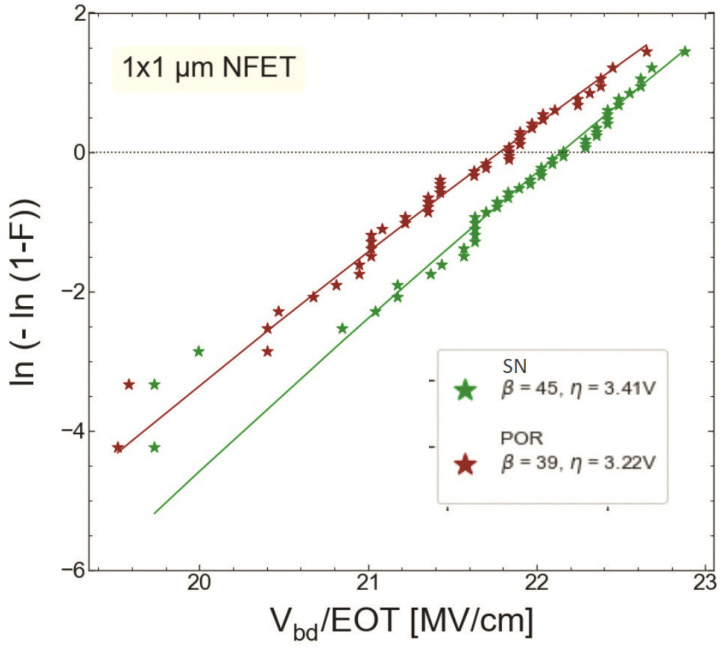
Weibit plots for breakdown fields extracted for nMOSFETs grown by POR and SN processes.

We compared HBD experimental data sets obtained only for MOSFETs with the targeted thickness of 1.9 nm fabricated by the POR (S #1) and with the SN step (S #2). Figure 15 and Figure 16 show the gate current $I_{g}$ as a function of stress gate voltage $V_{g}$ for n-channel transistors from wafers S #1 and S #2, respectively. Gate voltage values corresponding to HBD events ($V_{\mathrm{BD}}$) were extracted and binned into distributions, which are represented as Weibit plots in Figure 17. One can observe that the Weibull distribution of ($V_{\mathrm{BD}}$) extracted for the transistor fabricated by the SN process is shifted by ∼0.2 V towards higher voltages compared to that obtained for the POR MOSFET. However, these samples have slightly different EOT values; therefore, the Weibull distribution of the breakdown field $E_{\mathrm{BD}}$ (the ratio between $V_{\mathrm{BD}}$ and EOT) shown in (Figure 18) appears to be more informative. From Figure 18, we conclude that the POR nMOSFET has lower breakdown fields compared to their counterparts fabricated with the softer nitridation step.

**Figure 19:**
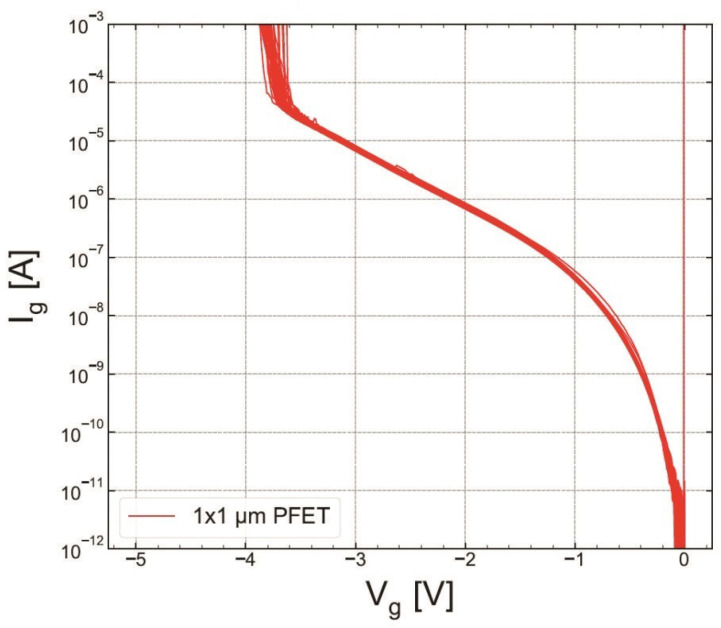
S #1 (POR), pMOSFETs: gate current $I_{g}$ as a function of stress voltage $V_{g}$ recorded during RVS HBD measurements conducted on the POR nMOSFET (sample S #1).

**Figure 20:**
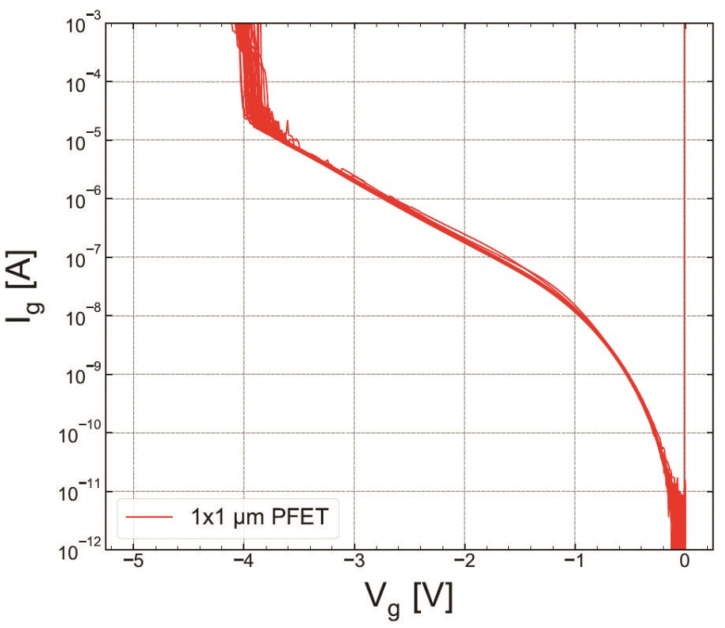
The same as Figure 19 but for S #2 (SN).

**Figure 21:**
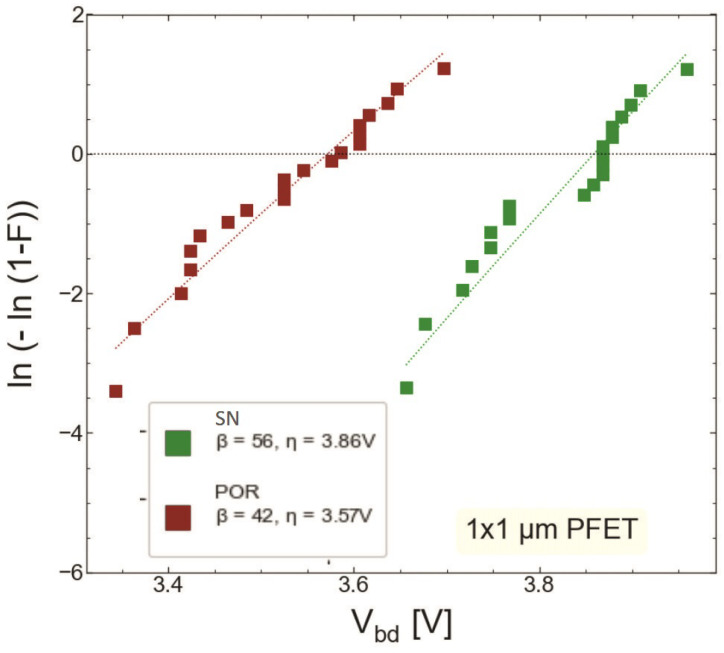
Weibit plots of breakdown voltages for the pMOSFETs fabricated by the POR (red symbols) and SN (green symbols).

**Figure 22:**
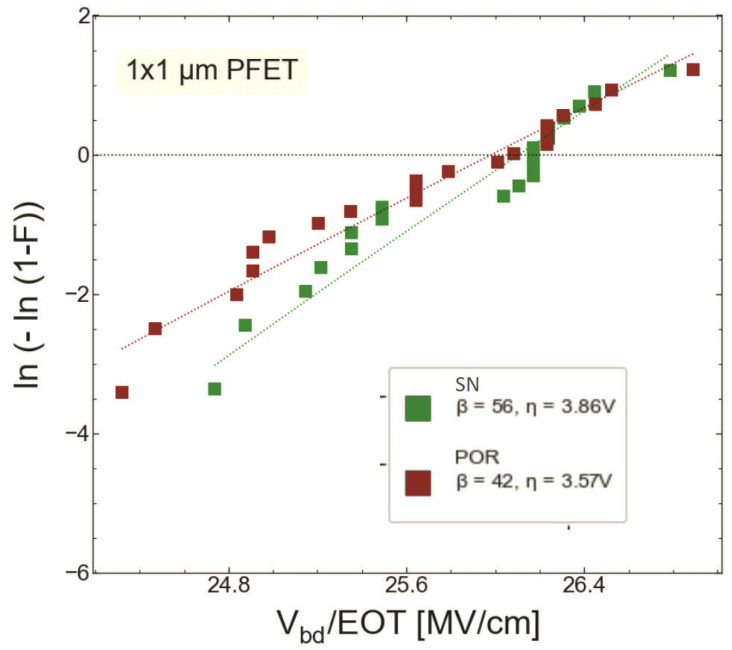
Weibit plots for breakdown fields extracted for pMOSFETs grown by POR and SN processes.

As for RVS-HBD in pMOSFETs, $I_{g}\left(V_{g}\right)$ dependencies for both POR and SN samples are shown in Figure 19 and Figure 20, respectively. The extracted $V_{\mathrm{BD}}$ (Figure 21) and $E_{\mathrm{BD}}$ (Figure 22) distributions confirm again that the SN sample has superior HBD robustness. However, the shift of the $E_{\mathrm{BD}}$ distribution towards higher $E_{\mathrm{BD}}$ values for the SN sample with respect to that extracted for the POR sample is not significant.

### 4.3. Interpretation of the Results

**Figure 23:**
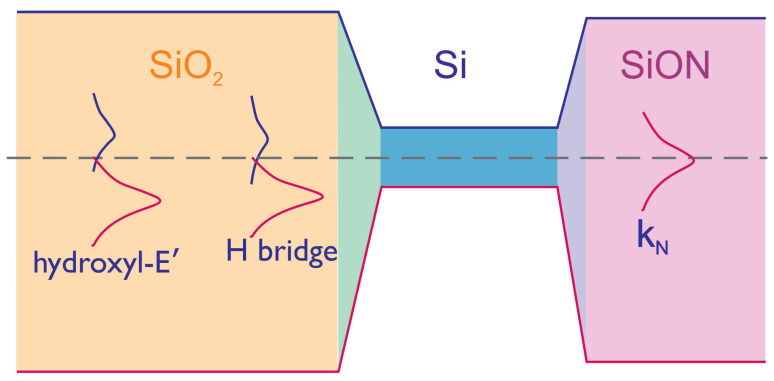
Schematically represented charge transition levels of traps responsible for NBTI in ${\mathrm{SiO}}_{2}$-based MOSFETs—hydroxyl-E’ centers and H bridges. Both form acceptor-like states close to the edge of the Si conduction band as well as donor-like states placed below the valence band of Si. In FETs with SiON/Si${}_{3}$N${}_{4}$ layers, another type of defects, namely k${}_{N}$ centers, govern BTI. They form the defect band with a centroid close to the mid-gap of Si.

According to the current understanding of the microscopic nature of BTI in ${\mathrm{SiO}}_{2}$-based transistors, traps responsible for this detrimental phenomenon are the hydrogen bridges and the hydroxyl-E’ centers [63]. These traps form two bands in the band gap of ${\mathrm{SiO}}_{2}$: the band of acceptor-like states, which is situated in the upper half of the band gap, and the band of donor-like states in the lower half of the band gap [62,64]; see Figure 23. The centroid of the band of donor-like traps is situated below the Si valence band edge; therefore, the charge exchange between these traps and the valence band results in NBTI (and its recovery). On the other hand, the energetical levels of acceptor-like traps are close to the conduction band of Si, but their density-of-states is low; therefore, they provide a negligible contribution to PBTI (this behavior is consistent with experimental data reported in this work). As for traps responsible for BTI in SiON/Si${}_{3}$N${}_{4}$-based MOSFETs, they were identified as k${}_{N}$ centers with the corresponding trap density-of-states featuring a peak close to the center of the Si band gap [64,65,66,67]. As a result, they are more easily accessible for holes, thereby making the NBTI stronger in MOSFETs with SiON/Si${}_{3}$N${}_{4}$ layers compared to their ${\mathrm{SiO}}_{2}$-based counterparts (under the same stress conditions). Therefore, an increasing N content in the SiON film leads to the “transition” from the hydrogen bridges and hydroxyl-E’ centers to k${}_{N}$ centers and hence more prominent NBTI. This picture is consistent with the explanation of stronger NBTI typical for SiON-based devices compared to MOSFETs with ${\mathrm{SiO}}_{2}$ as the gate dielectric given by Reisinger et al. [35,68].

As for HBD, this event is a terminal stage of TDDB when a local concentration of generated defects is so high that these defects form a percolation path (with ohmic conductivity) [12,50]. Recent studies suggested that the microscopic mechanism underlying defect creation during TDDB/HBD is generation of O vacancies driven by the double trapping of electrons at wide angle O-Si-O sites (defect precursors) [69,70]. Although we are not aware of any papers studying the structure of TDDB-related defects and their precursors at a varying concentration of N in SiON, we can speculate that, at a higher N concentration, the energetical position of defect precursors becomes more favorable for phonon-assisted tunneling, thereby giving rise to defect creation and thus HBD at lower fields/voltages.

## 5. Conclusions

We compared bias temperature instability and hard breakdown characteristics of planar transistors with SiON layers with different nitrogen content. To accomplish this task, we attracted MOSFETs fabricated using processes with 1000 W (POR) and 900 W (SN) powers of radio frequency plasma nitridation; consequently, corresponding SiON films were exposed to different doses of N. In addition to this, we also used samples which—although fabricated by the same process flow—have different targeted thicknesses of SiON layers, thereby leading to different values of the N content. Nitrogen incorporated in gate oxides is known to result in positive charges distributed throughout the dielectric layer; these charges result in a threshold voltage shift towards lower $V_{t}$ values. The threshold voltages values of pristine devices are consistent with the N content estimated based on the process flow details.

All MOSFETs were subjected to ramped voltage stress to study the BTI and HBD behavior of these transistors. In case of BTI, p-channel MOSFETs were stressed in the NBTI regime and nMOSFETs were subjected to PBTI. Regarding HBD, stress measurements applied to n- and p-channel MOSFETs were carried out at positive and negative gate voltages, respectively.

NBTI data demonstrated a pronounced trend—NBTI reliability is correlated with the N concentration of SiON layers, i.e., devices with a lower N content demonstrated a superior NBTI behavior (smaller $\Delta V_{t}$ shifts and longer TTF values). PBTI appeared to be negligibly weak in both devices with low and high N content. In the case of HBD, we compared MOSFETs with the same targeted thickness of SiON layers but fabricated using POR and SN processes. The latter samples have a lower N concentration; their Weibull distribution of the breakdown electric field is shifted towards higher values with respect to that obtained for MOSFETs with a high N content. Therefore, similar to BTI, transistors with a smaller N concentration feature a superior HBD behavior.

The aggravation of BTI in MOSFETs with a higher N concentration can be explained within the paradigm that in ${\mathrm{SiO}}_{2}$-based (or SiON with a low N dose) transistors, traps responsible for BTI are the hydrogen bridges and hydroxyl-E’ centers, while in transistors with SiON/Si${}_{3}$N${}_{4}$ as the gate dielectric, BTI originates from k${}_{N}$ centers, which have different energy positions and therefore are more accessible by carriers. Regarding HBD, this event corresponds to such a high concentration of traps, that they form a percolation path and the dielectric film loses its insulating properties. The physical mechanism underlying the defect creation is related to double electron trapping at a wide angle O-Si-O bond, and we expect that at a higher N concentration, energetical positions of these precursor sites are shifted towards values more favorable for tunneling.

It is worth emphasizing that there is no consensus in literature regarding the impact of nitridation on BTI behavior; therefore, our result is consistent with findings of those groups who reported aggravation of BTI in strongly nitrided SiON films. Regarding the influence of nitridation on HBD, there are the very limited number of papers dealing with this topic; our contribution additionally confirms that HBD occurs at lower electric fields in devices with a higher N content.

## Figures and Tables

**Table 1:** Equivalent oxide thickness and threshold voltage values of samples used for BTI and HBD studies shown for n- and p-channel MOSFETs. EOT data are acquired using capacitance-voltage measurements, while for $V_{t}$ extraction, the maximum transconductance method (at the drain voltage $V_{d}$ of +0.05 and −0.05 V for n- and pMOSFETs, respectively) was employed. The last column shows the N content rank. A higher rank corresponds to a higher concentration, i.e., ‘1’ corresponds to the lowest N content and ‘4’ to the highest N concentration.

Sample	Details	EOT, nMOS	EOT, pMOS	$V_{t}$, nMOS	$V_{t}$, pMOS	N Content Rank
S #1	POR	1.48 nm	1.37 nm	0.23 V	−0.22 V	3
S #2	SN	1.54 nm	1.48 nm	0.28 V	−0.17 V	1
S #3	POR + 2 Å	1.78 nm	1.66 nm	0.29 V	−0.20 V	2
S #4	SN − 2 Å	1.49 nm	1.34 nm	0.13 V	−0.27 V	4
S #5	SN − 2 Å	1.47 nm	1.28 nm	0.13 V	−0.27 V	4

## Data Availability

The data presented in this study are available on request from the corresponding author.

## Abbreviations

The following abbreviations are used in this manuscript:

BTI	Bias temperature Instability
CVS	Constant Voltage Stress
eMSM	extended Measure–Stress–Measure (technique)
EOL	Equivalent Oxide Thickness
HBD	Hard Breakdown
HCD	Hot-Carrier Degradation
MOSFET	Metal–Oxide–Semiconductor Field Effect Transistor
NBTI	Negative Bias Temperature Instability
PBTI	Positive Bias Temperature Instability
POR	Process of Reference
RF	Radio Frequency
RVS	Ramped Voltage Stress
SN	Soft Nitridation
TDDB	Time-Dependent Dielectric Breakdown
